# Association of Prepregnancy Obesity and Remodeled Maternal-Fetal Plasma Fatty Acid Profiles

**DOI:** 10.3389/fnut.2022.897059

**Published:** 2022-05-16

**Authors:** Hai-Tao Yu, Wen-Hui Xu, Yi-Ru Chen, Ye Ji, Yi-Wei Tang, Yue-Ting Li, Jia-Yu Gong, Yi-Fei Chen, Guo-Liang Liu, Lin Xie

**Affiliations:** ^1^Department of Nutrition and Food Hygiene, School of Public Health, Jilin University, Changchun, China; ^2^Experimental Teaching Center for Preventive Medicine, School of Public Health, Jilin University, Changchun, China

**Keywords:** pre-pregnancy BMI, overweight/obesity, fatty acids, cord blood, DHA

## Abstract

**Background:**

Fatty acids, especially polyunsaturated fatty acid (PUFA), are found abundantly in the brain and are fundamental for a fetus's growth. The fatty acid profiles of mothers and fetuses may be affected by maternal prepregnancy body mass index (pre-BMI), thus affecting fetal growth and development.

**Methods:**

A total of 103 mother-fetus pairs were divided into overweight/obese (OW, *n* = 26), normal weight (NW, *n* = 60), and underweight (UW, *n* = 17) groups according to pre-BMI. Fatty acid profiles in maternal and umbilical cord plasma were analyzed by gas chromatography.

**Results:**

The infant birth BMI *z*-score of the OW group was higher than that of the NW and UW groups (*p* < 0.05). The OW mothers had significantly higher plasma n-6 PUFA and n-6/n-3, but lower docosahexaenoic acid (DHA) and n-3 PUFA (*p* < 0.05). In cord plasma, the proportions of DHA and n-3 PUFA were lower in the OW group (*p* < 0.05), whereas the n-6/n-3 ratio was higher in the OW group (*p* < 0.05). The pre-BMI was negatively correlated with cord plasma DHA in all subjects (*r* = −0.303, *p* = 0.002), and the same negative correlation can be observed in the OW group (*r* = −0.561, *p* = 0.004), but not in the NW and UW groups (*p* > 0.05). The pre-BMI was positively correlated with cord plasma n-6/n-3 in all subjects (*r* = 0.325, *p* = 0.001), and the same positive correlation can be found in the OW group (*r* = 0.558, *p* = 0.004), but not in NW and UW groups (*p* > 0.05).

**Conclusions:**

Maternal pre-BMI was associated with the maternal-fetal plasma fatty acid profiles, whereas the adverse fatty acid profiles are more noticeable in the prepregnancy OW mothers.

## Introduction

Obesity is a major public health problem worldwide. The prevalence of obesity in women of reproductive age is increasing. Obesity during pregnancy is associated with adverse pregnancy outcomes and adverse offspring health-related issues such as fetal overgrowth (1–3). Moreover, maternal prepregnancy obesity has been reported to affect neurodevelopment of offspring (4). Also, intrauterine nutrient exposure of the fetus may have a long-term influence on growth and development, including the possibility of some future metabolic diseases (5). These effects might be related to disturbed polyunsaturated fatty acid (PUFA) compositions in mothers and fetuses. PUFA are involved in the composition of cell membrane phospholipids and are vital in brain and retina development (6, 7). Previous studies in obesity demonstrated a disturbed fatty acid profile (8). Exposure to adverse fatty acids profile *in utero* may affect fetal brain development (9).

Currently, studies on the influence of maternal obesity on the metabolism of infant fatty acids mainly focus on lactation, such as the influence of breast milk fatty acid composition on infant growth and development (10–15). However, the central nervous system undergoes a growth spurt from the third trimester of pregnancy to 18 months postnatally (16). Therefore, the nutritional status of maternal fatty acids during the last trimester of pregnancy is also very important for fetal growth and development. There are few studies on the effect of prepregnancy body mass index (pre-BMI) on the level of fetal fatty acids (17). Most studies focus on the relationship of maternal dietary fatty acids and the fetal development (18, 19). However, many factors affect the absorption of dietary fatty acids and their transport to the fetus, such as genetic factors (20) and the mother's physiological state (21). Genetic variations of the fatty acid desaturase (FADS) and elongase enzymes affect PUFA production. However, it has been reported that maternal BMI changes the effect of different genotypes on fatty acid levels, wherein overweight women were less affected by FADS genetic variants (22). Therefore, maternal pre-BMI may affect maternal and fetal fatty acid metabolism. Consequently, this study aimed to systematically analyze the plasma fatty acid profiles in prenatal and umbilical cord blood in mothers with different pre-BMIs. The association between pre-BMI and maternal and fetal plasma fatty acids was explored.

## Methods

### Subjects

Mother-fetus pairs were recruited from 2019 to 2020 at the first hospital of Jilin University, Changchun, China. This study adhered to the guidelines laid down in the Declaration of Helsinki, and all procedures involving human subjects were approved by the Chinese Clinical Trial Registry (ChiCTR2000034179). Written informed consent was obtained from all subjects. Maternal and infantile demographics and physiology characteristics were obtained through questionnaire and hospital medical records. In brief, 103 dyads of healthy pregnancy women were recruited at the last time visit to obstetric clinic before delivery and classified according to pre-BMI, namely, normal weight (NW, BMI = 18.5–23.99 kg/m^2^, *n* = 60), overweight/obese (OW, BMI ≥ 24 kg/m^2^, *n* = 26), and underweight (UW, BMI < 18.5 kg/m^2^, *n* = 17). The inclusion criteria were maternal age ≥20 years and ≤40 years, singleton pregnancy, gestation duration ≥37 weeks, and newborn health (Apgar>8). Women were excluded if they were suffering from metabolic diseases (e.g., prepregnancy and gestational diabetes mellitus), HIV-infected disease, pulmonary tuberculosis, other acute infectious diseases, severe heart disease and renal disease, and were taking drugs that affect nutrient metabolism, and infants with congenital and hereditary diseases were excluded. The information on maternal age, pre-BMI, antenatal BMI, gestational weight gain, gestation duration, infant anthropometry, and sex was investigated by questionnaire and medical record.

### Dietary Assessment

A valid food frequency questionnaire (FFQ) was carried out by the investigators who were trained uniformly. We gave each participant a face-to-face interview and a semistructured FFQ. Based on 7 different food groups (e.g., meat, eggs, poultry, fish and seafood, fruits, vegetables, milk, etc.,) and 54 different food categories combined with food pictures and standard food mold for food weight evaluation, the questionnaire was used to assess the dietary intake of enrolled subjects during the last trimester. It included specific questions about the cooking styles, cooking oil types, and dosages sources containing docosahexaenoic acid (DHA, C22:6n-3), such as freshwater fish, seafood, and canned tuna. The intake and frequency of food categories per day/week/month were recorded. To better understand the data of DHA intake, in the interview, we also asked participants about the DHA supplement's brand and daily doses. The investigators checked the content of DHA in supplements and calculated the daily doses of DHA. The questionnaire was improved and used by our group to assess the dietary intake, especially fatty acids (23). The intake of energy and five kinds of PUFA dietary intakes were calculated according to the food composition table (24). Finally, 79 questionnaires were collected, 24 subjects did not complete the questionnaire well (OW = 5, NW = 12, UW = 7).

### Sample Collection

A total of 5 ml maternal blood samples after an overnight fast was collected either on the morning of admission for surgery in case of primary cesarean sections or at the last visit to the obstetric clinic, no longer than 3 days before delivery. Then, 5 ml cord blood samples were collected at delivery by a maternity nurse. All blood samples were collected in EDTA tubes and centrifuged at 3,500 rpm for 15 min to separate plasma, and stored at −80°C until fatty acids analysis.

### Plasma Fatty Acid Analysis

A direct methylation procedure was performed on 100 μl of plasma. Then, 100 μl plasma, 100 μl C17:0 internal standard solution (5 mg/ml), and 600 μl methanol were mixed and vortexed for 30 s, and centrifuged for 5 min at 900 × *g*. The methanol phase was taken to another glass centrifuge tube, mixed with 25 μl sodium methoxide solution, and the solution was mixed for 3 min at room temperature. Also, 75 μl methanol hydrochloride solution was added to terminate reaction. Then, 300 μl *n*-hexane was added, and the mingled solution was mixed for 30 s to extract fatty acid methyl esters (FAMEs), then the upper *n*-hexane phase was transferred to a new glass centrifuge tube. The extraction was blown to dry by nitrogen, and 50 μl *n*-hexane containing 2 g/L butylated hydroxytoluene was added to dissolve the residue for gas chromatography (GC) analysis. The concentrations of 36 plasma fatty acids (μg/ml) were determined in relation to the peak area of internal standard. Each plasma fatty acid was expressed as a percentage of the total 36 fatty acid concentrations measured.

Plasma FAMEs were detected by using GC-2010Plus gas chromatography (Shimadzu Corp., Kyoto, Japan). The GC was equipped with a SP-2560 capillary column (100 m × 0.25 mm × 0.20 μm; Supelco, Bellefonte, PA). The chromatographic conditions were, namely, high-purity nitrogen was the carrier gas (linear velocity: 1 ml/min), split ratio was 1:50, and the injection volume was 1 μl; the initial temperature of the column box was set at 140°C, held for 5 min, and then the temperature rose to 260°C at the rate of 4°C /min, held for 20 min; the temperature of flame ionization detector was set at 280°C; the flow rate of hydrogen was 40 ml/min, and airflow rate was 500 ml/min. Shimadzu lab solutions chromatography workstation software was used to record the chromatogram, retention time, and peak area. The fatty acid concentrations were calculated by comparing the peak area of internal standard. The levels of plasma fatty acids are expressed as the percentage of total fatty acid (%).

### Desaturase Enzyme Indices

Linoleic acid (LA; C18:2n-6) is the precursor of n-6 PUFA, whereas α-linolenic acid (ALA; C18: 3n-3) is the precursor of n-3 PUFA (25). The process by which PUFA are synthesized from LA and ALA in the human body involves Δ6 desaturase (D6D, LA to γ-linolenic acid, ALA to stearidonic acid) and Δ5 desaturase [D5D, dihomo-γ-linolenic acid (DGLA) to arachidonic acid (AA), eicosatetraene acid to eicosapentaenoic acid (EPA)]. Product-to-precursor ratios have been used to represent enzyme indices (26–28). In this study, the D6D index was estimated by DGLA (20:3n-6)/LA (18:2n-6), the D5D index was estimated by AA (20:4n-6)/DGLA (20:3n-6), and the ratio for estimation of the elongase activity was C18:1/C16:1 (29).

### Statistical Analysis

The mean and standard error of mean (SEM) were used for normal variables, and quartile was used to describe continuous variables of non-normally distribution. The minimum sample size was calculated according to relevant literature (30). One-way ANOVA and Kruskal-Wallis *H* test were used for the analysis of anthropometric parameters and fatty acids. Chi-square test was used for nonquantitative variables. Pearson and Spearman were used to analyze the association between maternal parameters and infant parameters. Multivariable linear regression was used to analyze the associations between maternal parameters and infant DHA. In linear regression model 1, all significant maternal factors were included, and maternal age and gestation weight gains were adjusted to assess the relative importance without the influence of dietary fatty acids. In linear regression model 2, dietary fatty acids were adjusted. Statistical significance was set at *p* < 0.05, data were analyzed using SPSS 24.0 software package (SPSS Inc., Chicago, IL, USA), and figures were drawn using R soft.

## Results

### Anthropometric and Demographic Characteristics

A total of 103 women enrolled and completed the study, and maternal-fetal anthropometric and demographic characteristics are presented in Table 1. As expected, maternal prepregnancy and antenatal BMIs were significantly higher in the OW group than the other two groups (*p* < 0.001). The gestational weight gains were significantly lower in the OW group than the other two groups (*p* < 0.001). Infants born to OW and NW mothers presented significantly higher birth weight than infants born to UW mothers (*p* = 0.01, *p* = 0.03). Infants born to OW mothers presented significantly higher birth BMI z-score than NW and UW mothers (*p* = 0.034, *p* = 0.012). Infants born to NW mothers presented higher birth length than infants born to UW mothers (*p* = 0.011).

**Table 1:** Anthropometric and demographic characteristics of mothers and infants according to maternal prepregnancy BMI categories.

	**OW (*n =* 26)**	**NW (*n =* 60)**	**UW (*n =* 17)**
Maternal age (years)	30.54 ± 0.75	30.88 ± 0.37	29.35 ± 0.69
Pre-pregnancy BMI (kg/m^2^)	**27.28 (25.39, 27.78)^a^^c^**	**21.06 (20.23, 22.04)**	**17.89 (16.79,18.08)^b^**
Weight gain (kg)	**13.08** **±1.29^a^^c^**	**18.25** **±0.77**	**20.71** **±1.39**
Antenatal BMI (kg/m^2^)	**32.18** **±0.47^a^^c^**	**28.16** **±0.32**	**23.82** **±1.60^b^**
Gestational duration (weeks)	38.91 ± 0.23	39.18 ± 0.20	39.25 ± 0.26
**Delivery modes**			
Cesarean (*n*, %)	22 (84.62)	41 (73.21)	11 (68.75)
Vaginal delivery (*n*, %)	4 (15.38)	15 (26.79)	5 (31.25)
Dietary fatty acids intake^*^			
LA (g/d)	18.89 ± 1.01	18.49 ± 0.72	18.65 ± 1.98
ALA (g/d)	1.98 ± 0.14	1.86 ± 0.11	2.02 ± 0.30
AA (mg/d)	36.54 (28.70, 60.34)	37.75 (28.80, 61.31)	49.70 (23.28, 99.09)
EPA (mg/d)	9.37 (4.05, 17.61)	15.72 (3.87, 53.28)	15.19 (6.23, 18.58)
DHA (mg/d)	18.35 (7.59, 207.16)	43.44 (7.64, 202.61)	18.23 (4.16, 32.26)
Dietary energy intake(kcal/d)^*^	2,149.10 ± 124.55	2,332.84 ± 95.87	2,291.89 ± 153.24
**Infant sex**			
Male (*n*, %)	13 (50.00)	39 (66.10)	10 (58.82)
Female (*n*, %)	13 (50.00)	20 (33.90)	7 (41.18)
Infant birth weight(g)	**3,559.36** **±95.06^c^**	**3,474.07** **±46.26**	**3,240.59** **±82.22^b^**
Infant birth length (cm)	50.52 ± 0.37	**50.76** **±0.18**	**49.65** **±0.40^b^**
Infant birth BMI z-score	**0.46** **±0.19^a^^c^**	**0.03** **±0.10**	**−0.21** **±0.22**

*BMI, body mass index; OW, mothers with overweight or obese; NW, mothers with normal weight; UW, mothers with underweight; LA, linoleic acid; ALA, alpha-linolenic acid; AA, arachidonic acid; EPA, eicosapentaenoic acid; DHA, docosahexaenoic acid*.

*Data presented as mean ± standard error or P_50_ (P_25_, P_75_). Bold font indicates statistical significance at p < 0.05*.

a *OW vs. NW*.

b *NW vs. UW*.

c *OW vs. UW*.

* *24 maternal FFQ missing (OW = 5, NW = 12, UW = 7)*.

### Maternal Plasma Fatty Acid Profile

Maternal plasma fatty acids are listed in Table 2, and two kinds of fatty acids (C11:0, C23:0) were below the limit of quantification; the proportions of C4:0, C6:0, C8:0, C10:0, C12:0, C13:0, C20:0, C21:0, C22:0, C24:0, C14:1, C15:1, C17:1, C18:1(trans), C24:1 were lower than 0.10%, thus they are not presented in Table 2.

**Table 2:** The fatty acid profile in maternal plasma according to maternal prepregnancy BMI.

**Fatty acids (%)**	**OW (*n =* 26)**	**NW (*n =* 60)**	**UW (*n =* 17)**
**SFA**			
C14:0 (myristic acid)	0.34 (0.25, 0.50)	0.30 (0.24, 0.40)	0.32 (0.29, 0.44)
C15:0 (pentadecylic acid)	0.15 ± 0.02	0.15 ± 0.01	0.14 ± 0.02
C16:0 (palmitic acid)	30.18 ± 0.30	30.41 ± 0.19	29.88 ± 0.38
C18:0 (stearic acid)	9.93 ± 0.23	10.24 ± 0.17	10.14 ± 0.30
∑SFA	41.76 (39.66, 42.73)	40.48 (39.33, 43.21)	40.76 (39.63, 40.76)
**MUFA**			
C16:1 (palmitoleic acid)	0.57 ± 0.06	0.56 ± 0.04	0.65 ± 0.06
C18:1 (octadecanoenoic acid)	7.17 ± 0.23	6.85 ± 1.14	7.30 ± 0.33
C20:1 (gadoleic acid)	0.16 ± 0.02	0.15 ± 0.01	0.14 ± 0.01
C22:1 (brassidic acid)	0.22 ± 0.08	0.15 ± 0.04	0.19 ± 0.11
∑MUFA	8.64 ± 0.33	8.13 ± 0.19	8.79 ± 0.43
**PUFA**			
C18:2n-6 (trans linoleic acid, LA)	0.10 ± 0.03	0.17 ± 0.03	0.10 ± 0.03
C18:2n-6 (linoleic acid, LA)	**27.72** **±0.75^a^**	25.80 ± 0.48	25.92 ± 0.91
C18:3n-6 (gamma-linolenic acid, GLA)	0.13 ± 0.01	0.14 ± 0.02	0.12 ± 0.02
C18:3n-3 (alpha-linolenic acid, ALA)	0.35 ± 0.04	0.42 ± 0.05	0.39 ± 0.05
C20:2n-6 (eicosadienoic acid, EDA)	0.64 ± 0.08	0.56 ± 0.04	0.55 ± 0.04
C20:3n-6 (dihomo-γ-linolenic acid, DGLA)	3.14 ± 0.23	3.48 ± 0.10	3.22 ± 0.17
C20:3n-3 (eicosatrienoic acid)	0.79 ± 0.20	0.73 ± 0.12	0.76 ± 0.09
C20:4n-6 (arachidonic acid, AA)	10.81 ± 0.48	11.66 ± 0.29	11.63 ± 0.65
C22:2n-6 (docosadienoic acid)	0.13 (0.03, 0.76)	0.11 (0.05, 0.32)	0.16 (0.07, 0.31)
C20:5n-3 (eicosapentaenoic acid, EPA)	0.43 (0.34, 0.68)	0.52 (0.38, 0.67)	0.57 (0.42, 0.75)
C22:6n-3 (docosahexaenoic acid, DHA)	**6.07** **±0.31^a^^c^**	7.22 ± 0.21	7.12 ± 0.43
∑EFA	**28.08** **±0.75^a^**	26.22 ± 0.49	26.31 ± 0.89
∑n-6 PUFA	**42.90** **±0.43^a^**	41.86 ± 0.24	41.79 ± 0.60
∑n-3 PUFA	**7.16** **±0.39^a^**	8.27 ± 0.21	8.24 ± 0.51
AA/DGLA (D5D)	3.39 ± 0.20	3.49 ± 0.12	3.74 ± 0.26
DGLA/LA (D6D)	0.12 ± 0.01	0.14 ± 0.01	0.13 ± 0.04
AA/LA	0.41 ± 0.03	0.47 ± 0.02	0.47 ± 0.04
EPA/ALA	1.93 ± 0.46	1.94 ± 0.30	1.87 ± 0.32
C18:1/C16:1 (elongase activity)	11.69 (9.42, 18.15)	12.35 (9.79, 16.36)	12.64 (8.93, 14.59)
n-6: n-3 ratio	**6.57** **±0.48^a^^c^**	5.27 ± 0.14	5.39 ± 0.17

*BMI, body mass index; OW/OB, mothers with overweight or obese; NW, mothers with normal weight; UW, mothers with underweight. SFA, saturated fatty acid; MUFA, monounsaturated fatty acid; PUFA, polyunsaturated fatty acid; EFA, essential fatty acid*.

*Data presented as mean ± standard error or P_50_ (P_25_, P_75_). Bold font indicates statistical significance at p < 0.05*.

a *OW vs. NW*.

b *NW vs. UW*.

c *OB/OW vs. UW*.

OW mothers had significantly lower level of DHA compared with NW and UW mothers (*p* = 0.003, *p* = 0.042). Maternal plasma EFA and n-6 PUFA were significantly higher in the OW group than the NW group (*p* = 0.038, *p* = 0.048). The percentage of n-3 PUFA was lower in the OW group than the NW group (*p* = 0.011). No significant differences were observed in maternal plasma D5D, D6D, and elongase activity indices among the three groups. The indexes of D6D and AA/LA tended to a marginal decrease in OW mothers (*p* = 0.054, *p* = 0.084). The ratio of n-6/n-3 was higher in the OW group than the NW and UW groups (*p* = 0.001, *p* = 0.021).

### Umbilical Cord Plasma Fatty Acid Profile

Two kinds of fatty acids in cord plasma (C11:0, C23:0) were below the limit of quantification, and the proportions of C4:0, C6:0, C8:0, C10:0, C12:0, C13:0, C20:0, C21:0, C24:0, C14:1, C15:1, C17:1, C18:1(trans), C24:1, C18:2n-6(trans), C18:3n-3, C20:3n-3 were lower than 0.10%; therefore, they are not presented in Table 3.

**Table 3:** The fatty acid profile in fetal cord plasma according to maternal prepregnancy BMI.

**Fatty acids (%)**	**OW(*n =* 25)**	**NW(*n =* 60)**	**UW (*n =* 17)**
**SFA**			
C14:0 (myristic acid)	**0.37 (0.31, 0.44)^a^**	0.33 (0.30, 0.36)	**0.36 (0.31, 0.46)^b^**
C15:0 (pentadecylic acid)	**0.45 (0.09, 0.57)^a^**	0.55 (0.41, 0.66)	0.51 (0.13, 0.69)
C16:0 (palmitic acid)	25.72 ± 0.46	26.38 ± 0.23	26.17 ± 0.65
C18:0 (stearic acid)	12.59 ± 0.26	12.75 ± 0.33	13.11 ± 0.35
C22:0 (behenic acid)	0.43 ± 0.08	0.33 ± 0.02	0.31 ± 0.07
∑SFA	**40.78** **±0.62^c^**	41.31 ± 0.33	42.90 ± 1.04
**MUFA**			
C16:1 (palmitoleic acid)	1.22 ± 0.10	1.04 ± 0.07	1.10 ± 0.12
C18:1 (octadecanoenoic acid)	7.27 ± 0.21	7.30 ± 0.11	7.12 ± 0.35
C20:1 (gadoleic acid)	0.38 ± 0.13	0.39 ± 0.08	0.17 ± 0.08
C22:1 (brassidic acid)	0.68 (0.00, 2.51)	0.51 (0.00, 1.83)	0.16 (0.00, 0.45)
∑MUFA	**11.03 (9.43, 14.87)^c^**	9.76 (8.73, 12.98)	9.18 (8.51, 10.02)
**PUFA**			
C18:2n-6 (linoleic acid, LA)	10.40 ± 0.29	10.91 ± 0.40	10.59 ± 0.41
C18:3n-6 (gamma-linolenic acid, GLA)	0.19 ± 0.02	0.28 ± 0.08	0.15 ± 0.03
C20:2n-6 (eicosadienoic acid, EDA)	**0.89 (0.42, 2.27)^a^**	0.42 (0.20, 0.75)	0.44 (0.37, 0.70)
C20:3n-6 (dihomo-gamma-linolenic acid, DGLA)	5.52 (4.74, 6.35)	5.50 (4.95, 6.17)	5.42 (4.82, 6.15)
C20:4n-6 (arachidonic acid, AA)	19.83 ± 0.54	19.99 ± 0.35	20.07 ± 0.82
C22:2n-6 (docosadienoic acid)	0.54 (0.00, 2.18)	0.33 (0.00, 1.33)	0.00 (0.00, 0.81)
C20:5n-3 (eicosapentaenoic acid, EPA)	**0.41 (0.29, 0.50)^a^**	0.47 (0.36, 0.65)	0.51 (0.31, 0.67)
C22:6n-3 (docosahexaenoic acid, DHA)	**7.82** **±0.50^a^^c^**	8.83 ± 0.18	9.16 ± 0.53
∑ EFA	10.42 ± 0.29	10.94 ± 0.40	10.63 ± 0.43
∑ n-6 PUFA	38.90 ± 0.52	38.07 ± 0.33	37.95 ± 1.33
∑ n-3 PUFA	**8.27** **±0.53^a^^c^**	9.41 ± 0.19	9.72 ± 0.57
n-6: n-3 ratio	**4.95 (4.42, 6.29)^a^^c^**	4.15 (3.59, 4.72)	4.00 (3.34, 5.02)

*OW, mothers with overweight or obese; NW, mothers with normal weight; UW, mothers with underweight. SFA, saturated fatty acid; MUFA, monounsaturated fatty acid; PUFA, polyunsaturated fatty acid; EFA, essential fatty acid*.

*Data presented as mean ± standard error or P_50_ (P_25_, P_75_). Bold font indicates statistical significance at p < 0.05*.

a *OW vs. NW*.

b *NW vs. UW*.

c *OB/OW vs. UW*.

The proportions of C14:0 and C20:2n-6 in cord plasma were significantly higher in the OW group than that in the NW group (*p* = 0.034, *p* = 0.002). The proportions of C15:0, total saturated fatty acid (SFA), EPA, DHA, and n-3 PUFA were significantly lower in the OW group than that in the NW group (*p* = 0.041, *p* = 0.029, *p* = 0.038, *p* = 0.027, *p* = 0.017). MUFA was higher in the OW group than that in the UW group (*p* = 0.011). The ratio of n-6/n-3 was higher in the OW group than the NW and UW groups (*p* < 0.001, *p* = 0.001).

### Association of Maternal Parameters With Infant Parameters

**Figure 1:**
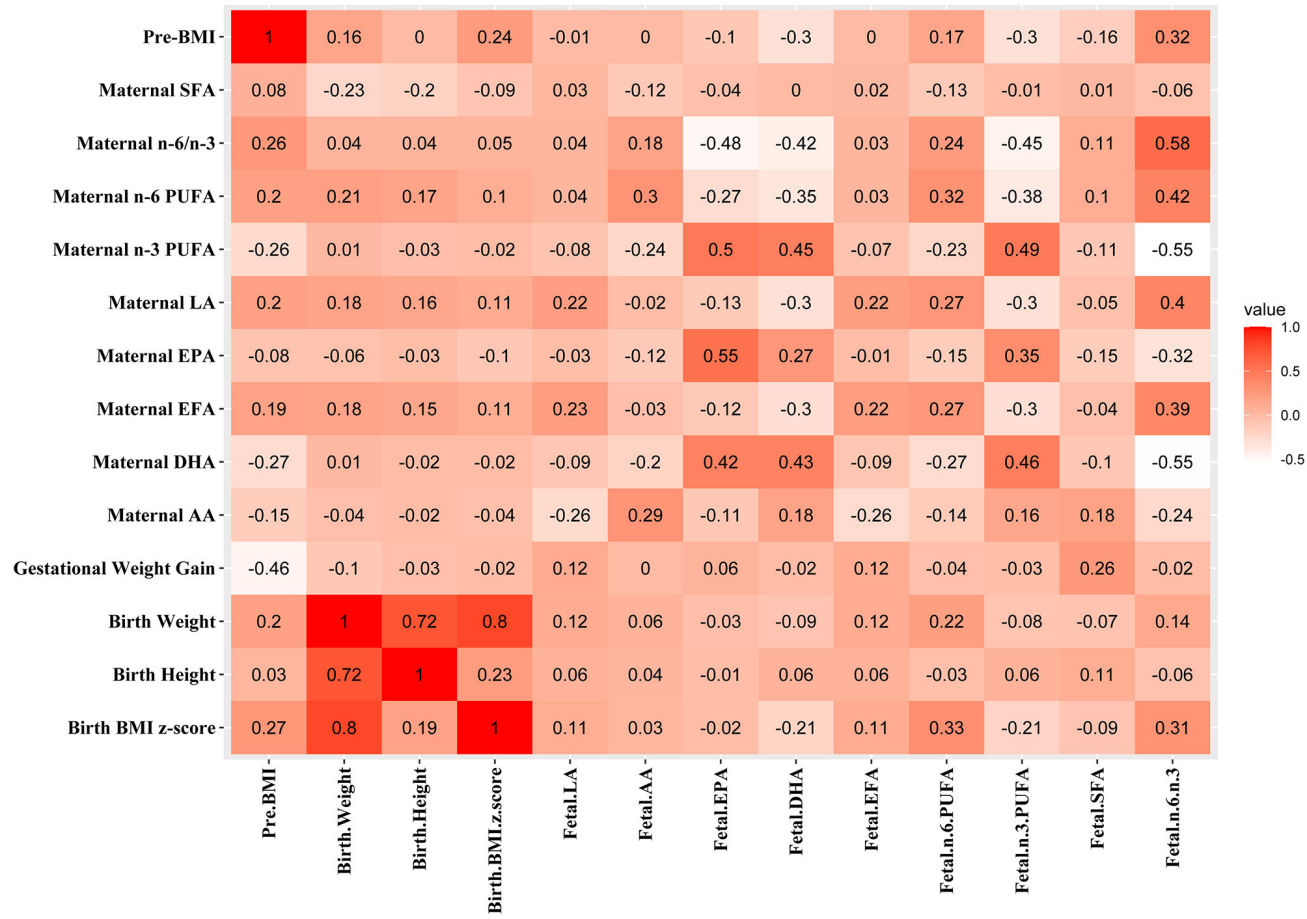
Associations between maternal parameters and fetal parameters. The correlation coefficients are shown in different colors. Red, positive correlation; white, negative correlation.

Associations between maternal parameters and the infantile parameters are shown in Figure 1. The pre-BMI was positively associated with maternal n-6 PUFA (*r* = 0.203, *p* = 0.039), n-6/n-3 PUFA (r = 0.258, *p* = 0.009), but the prepregnancy BMI was negatively associated with maternal DHA (*r* = −0.266, *p* = 0.007) and n-3 PUFA (*r* = −0.255, *p* = 0.009). Maternal pre-BMI was positively associated with infant birth BMI z-score (*r* = 0.274, *p* = 0.006) and cord plasma n-6/n-3 PUFA (*r* = 0.325, *p* = 0.001). Maternal pre-BMI was negatively associated with cord plasma DHA (*r* = −0.303, *p* = 0.002) and n-3 PUFA (*r* = −0.298, *p* = 0.002). The gestational weight gain was positively associated with cord plasma SFA (*r* = 0.261, *p* = 0.009). Maternal plasma EPA, DHA, and n-3 PUFA were positively correlated with cord plasma EPA, DHA, and n-3 PUFA (*p* < 0.05). Maternal plasma LA was positively associated with cord plasma LA, n-6 PUFA, and n-6/n-3, but negatively associated with cord plasma DHA and n-3 PUFA (*p* < 0.05).

### Subgroup Analysis of Association Between Maternal Pre-BMI and Fetal DHA, n-6/n-3

**Figure 2:**
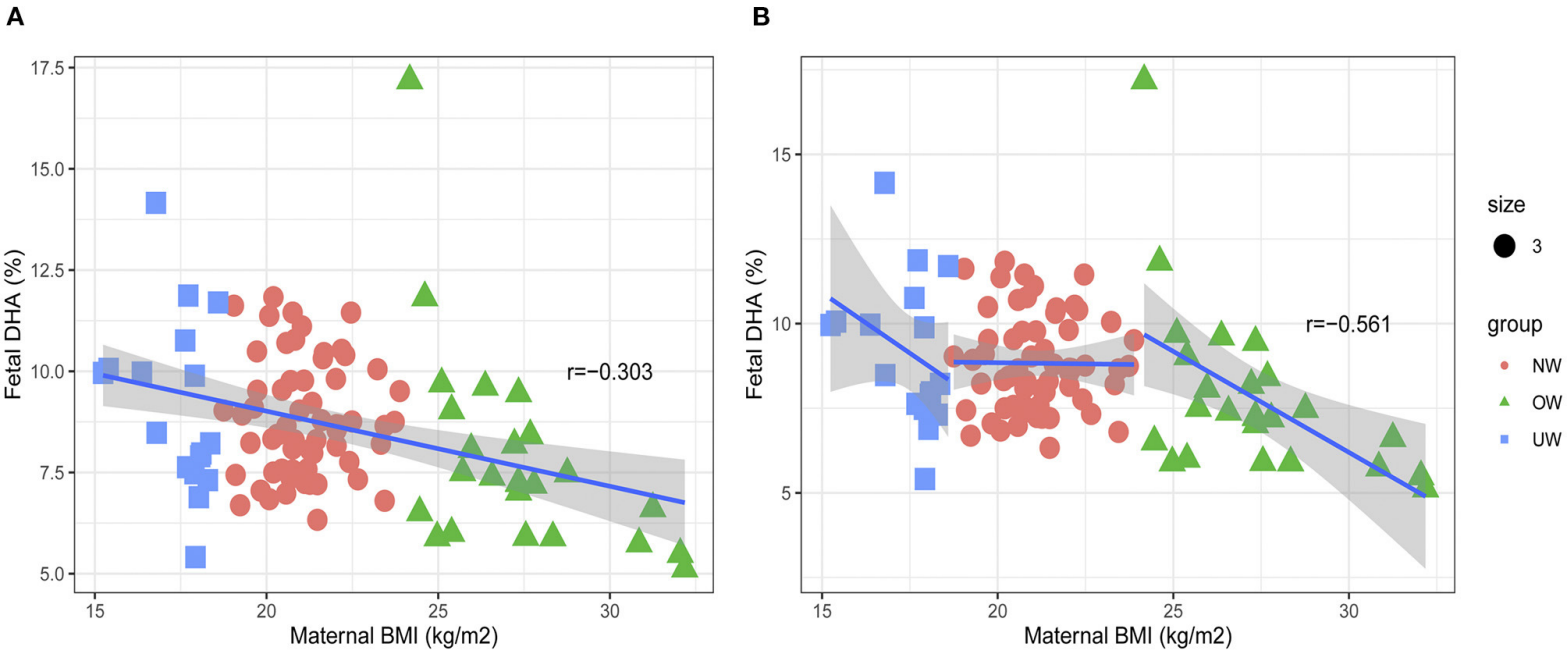
Associations between maternal pre-BMI and fetal DHA. **(A)** The association between maternal pre-BMI and fetal DHA in all subjects. **(B)** The association between maternal pre-BMI and fetal DHA in different groups.

The negative correlation was found between pre-BMI and cord plasma DHA in all subjects (Figure 2A). The subgroup analysis was carried out to explore the association of prepregnancy OW and cord plasma DHA (Figure 2B). The pre-BMI was negatively correlated with cord plasma DHA (*r* = −0.561, *p* = 0.004) in the OW group, but there were no correlations between pre-BMI and cord plasma DHA in NW (*r* = −0.012, *p* = 0.925) and UW subjects (*r* = −0.414, *p* = 0.098).

**Figure 3:**
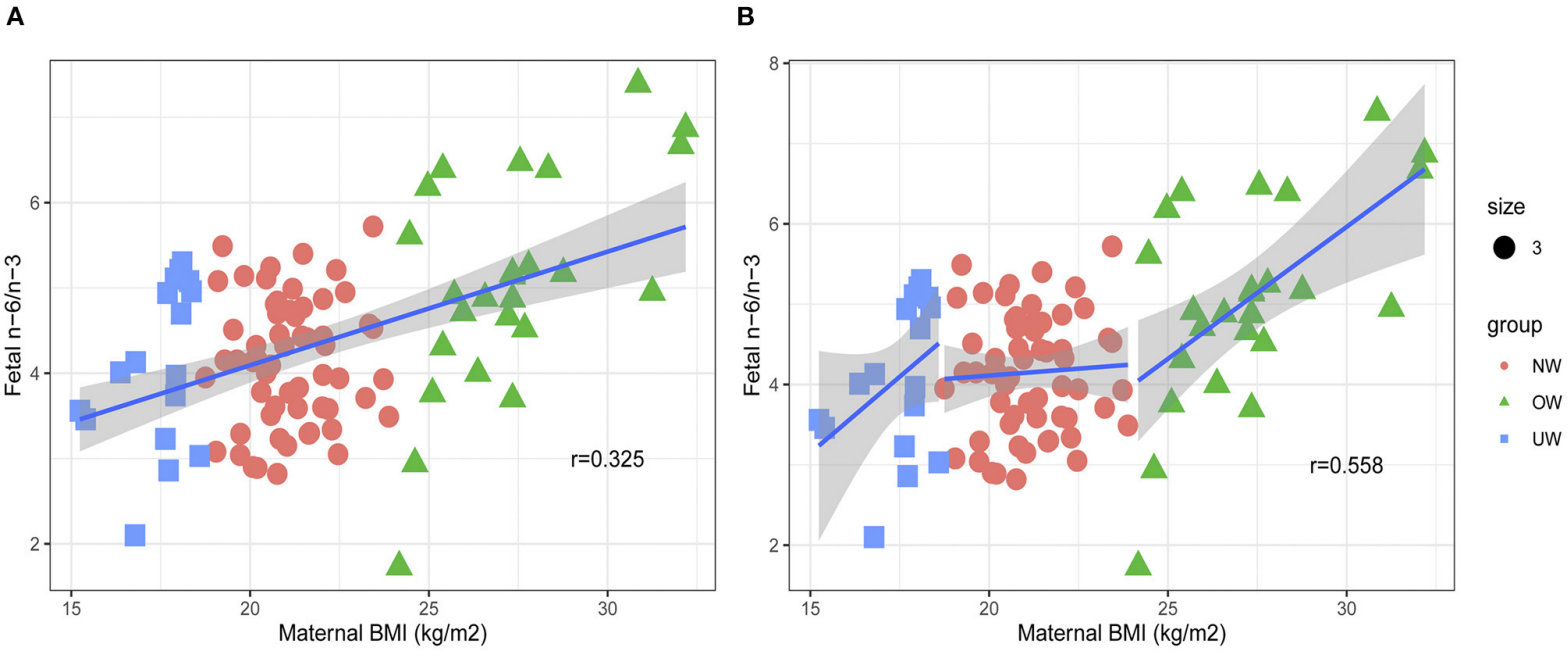
Associations between maternal pre-BMI and fetal n-6/n-3. **(A)** The association between maternal pre-BMI and fetal n-6/n-3 in all subjects. **(B)** The association between maternal pre-BMI and fetal n-6/n-3 in different groups.

The positive correlation was found between pre-BMI and cord plasma n-6/n-3 PUFA in all subjects (Figure 3A). Further subgroup analysis was carried out to explore the association of prepregnancy OW and cord plasma n-6/n-3 PUFA (Figure 3B). The pre-BMI was negatively correlated with cord plasma n-6/n-3 ratio (*r* = 0.558, *p* = 0.004) in the OW group, but there were no associations between pre-BMI and cord plasma n-6/n-3 in the NW (*r* = 0.041, *p* = 0.756) and UW groups (*r* = 0.439, *p* = 0.078).

### Multiple Linear Regression Analysis of Maternal Parameters and Fetal DHA

A number of confounder factors could influence the association between maternal parameters and fetal parameters. Multiple linear regression analysis was conducted to explore the direct correlation between maternal parameters and cord plasma DHA (Table 4). The correlations can be found between maternal pre-BMI, LA, EPA, DHA, EFA, n-6 PUFA, n-3 PUFA, and n-6/n-3 with cord plasma DHA in all subjects (Figure 1). Owing to collinearity, pre-BMI, maternal plasma LA, EPA, and DHA were included in the regression model. Multiple linear regression analysis demonstrated the associations between maternal pre-BMI and DHA with cord plasma DHA in all subjects (*p* < 0.05). In the subgroup analysis, the correlation between maternal pre-BMI and DHA with cord plasma DHA can be observed only in OW mothers (*p* < 0.05), but not in NW subjects.

**Table 4:** Linear regression analysis on the correlation of maternal parameters and fetal cord plasma DHA percentage.

**Maternal**	**All (*n* = 100)**		**OW (*n* = 25)**		**NW (*n* = 59)**		UW^*^(*n* = 16)	
**parameters**	**Beta**	***P***	**Beta**	***P***	**Beta**	***P***	**Beta**	***P***
**Model 1^a^**								
Pre-BMI	−0.330	**0.002**	–0.632	**<0.001**	–0.128	0.360	0.028	0.925
LA	–0.122	0.199	–0.386	**0.015**	–0.040	0.797	–0.113	0.738
EPA	0.123	0.185	–0.127	0.409	0.102	0.521	–0.510	0.267
DHA	0.290	**0.005**	0.584	**0.001**	0.163	0.314	0.652	0.157
**Model 2^b^**								
Pre-BMI	–0.327	**0.007**	–0.722	**0.001**	–0.081	0.572	–	–
LA	–0.114	0.305	–0.519	**0.022**	0.119	0.514	–	–
EPA	0.117	0.270	–0.220	0.267	–0.103	0.568	–	–
DHA	0.256	**0.029**	0.618	**0.004**	0.096	0.554	–	–
**Model 3^c^**								
Pre-BMI	–0.308	**0.013**	–0.717	**0.004**	–0.089	0.564	–	–
LA	–0.081	0.492	–0.456	**0.039**	–0.016	0.935	–	–
EPA	0.155	0.151	–0.158	0.509	0.035	0.844	–	–
DHA	0.252	**0.031**	0.577	**0.014**	0.163	0.359	–	–

*Associations were evaluated using linear regression analysis. Beta is corrected values after adjustment. p-Values < 0.05 are highlighted in bold*.

*Pre-BMI, prepregnancy body mass index; OW, mothers with overweight or obese; NW, mothers with normal weight; UW, mothers with underweight; LA, C18:2n-6, linoleic acid; EPA, C20:5n-3, eicosapentaenoic acid; DHA, C22:6n-3 docosahexaenoic acid*.

a *The multivariate regression analysis was adjusted for maternal age and gestation weight gains*.

b *The multivariate regression analysis was adjusted for maternal age, gestation weight gains, and dietary DHA. Due to the lack of dietary questionnaire, the sample size is reduced in model 2: all (n = 77), OW (n = 20), NW (n = 48), UW (n = 9)*.

c *The multivariate regression analysis was adjusted for maternal age, gestation weight gains, dietary DHA, and birth BMI z-score*.

* *The sample size was small, and regression analysis was not performed*.

### Multiple Linear Regression Analysis of Maternal Parameters and Cord Plasma n-6/n-3

Multiple linear regression analysis was conducted to explore the direct correlation between maternal parameters and cord plasma n-6/n-3 PUFA (Table 5). The correlations can be found between maternal pre-BMI, LA, AA, EPA, DHA, EFA, n-6 PUFA, n-3 PUFA, n-6/n-3, and cord plasma n-6/n-3 in all subjects (Figure 1). Owing to collinearity, finally pre-BMI, maternal AA, EPA, DHA, and n-6 PUFA were included in the regression model. Multiple linear regression analysis demonstrated the associations between pre-BMI and maternal DHA with cord plasma n-6/n-3 in all subjects (*p* < 0.05). In the subgroup analysis, maternal pre-BMI was associated with cord plasma n-6/n-3 in OW subjects (*p* < 0.05), but not in NW subjects (Table 5).

**Table 5:** Linear regression analysis on the correlation of maternal parameters and fetal cord plasma n-6/n-3.

**Maternal**	**All (*n* = 100)**		**OW (*n* = 25)**		**NW (*n* = 59)**		UW^*^(*n* = 16)	
**parameters**	**Beta**	***P***	**Beta**	***P***	**Beta**	***P***	**Beta**	***P***
**Model 1^a^**								
Pre-BMI	0.429	**<0.001**	0.686	**<0.001**	0.250	0.055	–0.146	0.483
AA	–0.139	0.080	–0.163	0.253	–0.123	0.329	0.080	0.714
EPA	–0.092	0.268	0.041	0.766	–0.235	0.111	0.827	**0.018**
DHA	–0.332	**0.001**	–0.420	**0.009**	–0.323	0.051	–0.905	**0.011**
n-6 PUFA	0.091	0.330	0.231	0.090	–0.171	0.296	0.435	0.074
**Model 2^b^**								
Pre-BMI	0.432	**<0.001**	0.697	**0.002**	0.355	**0.022**	–	–
AA	–0.111	0.235	–0.031	0.898	–0.116	0.452	–	–
EPA	–0.087	0.372	–0.014	0.939	–0.178	0.321	–	–
DHA	–0.282	**0.015**	–0.295	0.208	–0.263	0.166	–	–
n-6 PUFA	0.158	0.164	0.331	0.075	–0.127	0.518	–	–
**Model 3^c^**								
Pre-BMI	0.387	**0.001**	0.643	**0.008**	0.284	0.060	–	–
AA	–0.090	0.326	–0.172	0.521	–0.096	0.515	–	–
EPA	–0.139	0.156	–0.063	0.770	–0.184	0.286	–	–
DHA	–0.318	**0.006**	–0.326	0.199	–0.440	**0.031**	–	–
n-6 PUFA	0.070	0.598	0.316	0.116	–0.273	0.178	–	–

*Associations were evaluated using linear regression analysis. Beta is corrected values after adjustment. p-Values <0.05 are highlighted in bold*.

*Pre-BMI, prepregnancy body mass index; OW, mothers with overweight or obese; NW, mothers with normal weight; UW, mothers with underweight; AA, 20:4n-6, arachidonic acid; EPA, C20:5n-3, eicosapentaenoic acid; DHA, C22:6n-3 docosahexaenoic acid; PUFA, polyunsaturated fatty acid*.

a *The multivariate regression analysis was adjusted for maternal age and gestation weight gains*.

b *The multivariate regression analysis was adjusted for maternal age, gestation weight gains, and dietary total fatty acid. Due to the lack of dietary questionnaire, the sample size reduced in model 2: all (n = 77), OW (n = 20), NW (n = 48), and UW (n = 9)*.

c *The multivariate regression analysis was adjusted for maternal age, gestation weight gains, dietary total fatty acid, and birth BMI z-score*.

* *The sample size was small, and regression analysis was not performed*.

## Discussion

In this study, we observed that maternal prepregnancy BMIs were associated with maternal-fetal plasma fatty acid profiles. Maternal plasma DHA and n-3 PUFA were lower in the OW group, but n-6 PUFA and n-6/n-3 were higher. The cord plasma EPA, DHA, and n-3 PUFA were lower in the OW group, but n-6/n-3 was higher. Therefore, maternal prepregnancy obesity was associated with an adverse fatty acid profile in both mothers and fetuses. It had been reported that total n-3 PUFA was lower in the obese pregnant mothers, obesity was associated with an adverse fatty acids profile (1), and these results were in agreement with this study.

It had been reported that maternal plasma fatty acid was affected by dietary intake in addition to maternal metabolism (31). In this study, the dietary intake of fatty acids in the OW group did not differ from that in the NW group, but plasma LA was higher and DHA was lower in the OW group. A number of factors could account for this result. First, in addition to dietary intake, the endogenous synthesis of fatty acids also affects maternal fatty acid profile. It has been reported that maternal obesity affects the endogenous synthesis of fatty acids (32), further affecting umbilical cord blood fatty acid profile (33, 34). In this study, endogenous fatty acid synthesis involving the desaturase, the index of δ-6 fatty acid desaturase (AA/LA and DGLA/LA), tended to a marginal decrease in the OW group. The synthesis of subsequent products decreased, resulting in the underutilization of the substrate, then the substrate (LA) was higher in the OW group. In the human body, n-3 and n-6 PUFA share a set of fatty acid synthetase and elongase enzyme system, and there is a competitive relationship between the two pathways. The competition between fatty acid metabolic pathways may lead to changes in fatty acid composition not directly related to the diet. Secondly, methods of dietary assessment in this study require participants to recall their food consumption over the past 3 months. Therefore, there were a number of limitations that affect both accuracy and precision of dietary measurement. Respondents often underreport consumption, especially the OW participants (35). In addition, interviewer bias such as incorrect portion size estimations also account for the accuracy and precision of the dietary measurement. Relative intakes of individual fatty acids in the diet are therefore extremely difficult to estimate from reported dietary intakes. Thirdly, the lack of some dietary information also affected the accuracy of the results in this study. Besides, we performed this study in an inland city. Most pregnant women have the low dietary DHA intake (about 20 mg/day) (36), and part of pregnant women (about 30% in this study) intake DHA through a dietary supplement. Therefore, DHA dietary intake varies widely among individuals. Therefore, we observed the higher median of DHA intake in the OW group; it does not significantly differ from the other two groups. Although maternal plasma DHA was affected by both dietary and maternal metabolism, given these limitations, it is likely that the associations between dietary fatty intake and plasma fatty acids are limited by biases of dietary assessment in this study. Therefore, there has been considerable interest in using blood fatty acid composition as biological markers of fatty acids intake to reflect on dietary assessment (37). Compared to the dietary survey to assess maternal fatty acids intake (38), it is more accurate to investigate the relationship between maternal plasma fatty acids and fetal growth and development (39).

Previously, many researchers have focused on the relationship of prepregnancy obesity and fatty acids profile of breast milk, which could directly affect the infant's growth, body composition, and cognitive development (10, 11, 13). However, intrauterine development is also a key period. Fatty acids such as DHA are stored in maternal fat and are available when fetal fat accretion and brain growth increase exponentially during late pregnancy (40). Therefore, maternal fatty acids profile in this period is crucial for the fetus. This study investigated the association of maternal pre-BMI and maternal-fetal fatty acids profiles among different pre-BMI subgroups. There is a direct negative correlation between pre-BMI and cord plasma DHA and a direct positive correlation between pre-BMI and cord plasma n-6/n-3 in OW subgroup, but not in the NW and UW groups. We could conclude that the associations of maternal pre-BMI and maternal-fetal fatty acids profiles are not linear, and the statistical difference could be mainly attributed to the OW group. It is well known that maternal obesity is an important factor that affects the metabolism of fatty acids in mothers and fetuses (22). The body fat content causes different physiological statuses in mothers, influencing maternal metabolism and the placenta's transport function (41–43), consequently affecting fatty acid supply to the fetus.

It has been reported that maternal obesity modifies fatty acid profile, resulting in low n-3 and elevated n-6 PUFA levels in maternal circulation during pregnancy (44, 45). These modifications of the fatty acid profile are associated with a pro-inflammatory state and oxidative stress with short- and long-term consequences in the fetus and neonate. These changes confer a higher risk of developing obesity and its complications to the offspring (44). In this study, maternal plasma n-6/n-3 ratio was higher in OW mothers; correspondingly, the ratio of n-6/n-3 was higher in fetal cord plasma. Animal studies have found that maternal obesity may induce changes in the body fat composition or lead to obesity in offspring, which may be related to the increases in n-6/n-3 (46), and the decrease in n-6/n-3 had a protective influence on the development of offspring obesity (47). There are two critical periods in the fat development of infants, namely, before birth and the first year of life. The nutritional exposures during this time have permanent consequences on the regulation of body fat mass throughout life (12). It had been reported that a higher ratio of n-6/n-3 in umbilical cord blood was associated with a high subscapular skin-fold thickness at 3 years of age (48). Therefore, a fetus exposed to high levels of n-6/n-3 ratio may have an increased risk for childhood obesity and even adult obesity. These conclusions were in agreement with our results. Moreover, the pre-BMI was positively associated with birth BMI *z*-score and cord plasma n-6/n-3 in this study. It had been reported that infants born to mothers with prepregnancy obesity had a higher weight and length at birth (49). Birth weight and birth BMI *z*-score are the risk factors of offspring adult obesity and metabolism diseases; therefore, maternal prepregnancy obesity could be related to the offspring's growth and increase the risk of offspring obesity. In short, we found the associations of maternal prepregnancy obesity and adverse maternal-fetal fatty acid profiles, and the influence of maternal prepregnancy obesity on the offspring's growth and development requires further research. Besides, more DHA intake by diet is required in China, especially prepregnancy OW women.

A major limitation of this study was that only 103 mother-fetus pairs were collected; a larger sample size would provide further evidence to support the conclusions. The lack of some dietary information also affected the accuracy of the conclusion. Moreover, as this was a cross-sectional study, only the birth information of infants was collected. The offspring's follow-up growth and development information need to be collected in future studies. In conclusion, the pre-BMI was associated with the maternal-fetal plasma fatty acid profiles, whereas the adverse fatty acid profiles are more noticeable in the prepregnancy OW mothers.

## Data Availability Statement

The original contributions presented in the study are included in the article/Supplementary Material, further inquiries can be directed to the corresponding author.

## Ethics Statement

The studies involving human participants were reviewed and approved by Chinese Clinical Trial Registry (ChiCTR2000034179). Written informed consent to participate in this study was provided by the participants' legal guardian/next of kin.

## Author Contributions

H-TY: carrying out the study, analyzing the data, and writing the article. W-HX, Y-RC, and YJ: collecting sample. Y-WT, Y-TL, J-YG, and Y-FC: fatty acids detection. G-LL: methodology. LX: designing the study. All authors contributed to the article and approved the submitted version.

## Funding

This study was supported by the Graduate Innovation Fund of Jilin University (No. 101832020CX27) and Health Commission of Jilin Province (No. 2019J027).

## Conflict of Interest

The authors declare that the research was conducted in the absence of any commercial or financial relationships that could be construed as a potential conflict of interest.

## Publisher's Note

All claims expressed in this article are solely those of the authors and do not necessarily represent those of their affiliated organizations, or those of the publisher, the editors and the reviewers. Any product that may be evaluated in this article, or claim that may be made by its manufacturer, is not guaranteed or endorsed by the publisher.
